# The effects of hospital information system adoption on techno-stress and job satisfaction of nurses working in pediatric clinics

**DOI:** 10.1186/s12912-025-03371-8

**Published:** 2025-07-01

**Authors:** Bilge Sakrak, Aysel Kokcu Dogan

**Affiliations:** 1 Istanbul Medipol University, Institute of Health Sciences, Department of Nursing, Istanbul, Turkey; 2 Istanbul Medipol University, Faculty of Health Sciences, Department of Nursing, İstanbul, Turkey; 3 Istanbul Medipol University, Kavacık, Güney Kampüs, Göztepe Mah. Atatürk Cad. No: 40/16, Beykoz, İstanbul, 34810 Turkey

**Keywords:** Nurse, Hospital information system, Techno-stress, JOB satisfaction

## Abstract

**Background:**

We conducted this study to determine the effects of hospital information systems (HIS) adoption on techno-stress and job satisfaction among nurses working in pediatric clinics.

**Methods:**

This descriptive and correlational study included 190 nurses working in the pediatric clinics of a public hospital in Istanbul. Data were collected using the “Demographic Information Form”, “Hospital Information Systems Adoption Scale”, “Techno-stress Scale”, and “Minnesota Job Satisfaction Questionnaire”. Collected data were analyzed using the SPSS Windows 22.0 software package. Frequency and percentage were used to summarize the demographic characteristics of the nurses. Mean and standard deviation were used to examine the scores from the measurement tools. Correlations of scale sub-dimensions contributing to the overall scale or questionnaire scores were examined by Pearson’s correlation analysis and linear regression analyses. The t-test, one-way analysis of variance (ANOVA), and post hoc analyses (Tukey-LSD) were used to examine the differences in measurement tool scores by the demographic characteristics of nurses.

**Results:**

Of the participating nurses in the study; 68.9% were women, 46.8% were in the age range of 18–24 years, 62.1% were single, 66.3% had a bachelor’s degree or higher, 61.6% had been using computers for 3–10 years, and 54.7% had been using HIS for 0–3 years. A negative correlation was revealed between the adoption of HIS and perceived techno-stress among nurses, and the adoption of HIS was positively correlated with job satisfaction (*p* < 0.05).

**Conclusions:**

The adoption of HIS was highly effective in achieving reduced techno-stress levels and enhanced job satisfaction. The use of HIS will enable nurses to conduct documentation procedures faster than before, allowing nurses to allocate more time to spend with their pediatric patients to contribute to the improvement and promotion of pediatric health through the provision of a holistic professional care service dedicated to children.

**Clinical trial number:**

Not applicable.

## Introduction

Many factors, such as the quality of health services, reduction of medical errors, early diagnosis, appropriate treatment, reduction of high costs, patient satisfaction, and safety, have made the use of information technologies in health services mandatory [1]. The widespread use of information technologies improves the quality of care and strengthens clinical decision support processes. Healthcare information systems are defined as the integration of programs, software, methods, and instructions for the production, dissemination, and effective use of all kinds of information, ranging from the provision to the management of preventive, therapeutic, and rehabilitative healthcare services [2].

Although Hospital Information Management Systems (HIMS) are a new concept, they are an integrated software system that enables the management of all healthcare processes in hospitals in a digital environment [3]. The Healthcare Information and Management Systems Society (HIMSS) established the Electronic Medical Records Adoption Model (EMRAM) for hospitals in 2005 to enable organizations to conduct self-assessments of their levels of electronic medical record system adoption and usage and to allow them to benchmark against others. With the EMRAM scoring model, it is possible to rate hospitals for accreditation on a scale from HIMSS Zero (0) to seven (7) [4]. Hospitals that reach stages 6 and 7 of this roadmap, known as the adaptation process, are granted certification. The HIMSS 7 system, unlike other HIMS systems, is a system in which all patient and hospital data, especially decision support systems, are available in a digital environment, demonstrating that a healthcare organization has reached the highest level of innovation in the digital field [5]. Developing countries face numerous challenges in adopting new health technologies—such as lack of infrastructure, time, cost, qualified workforce, and absence of regulatory policies—compared to developed countries that have largely adopted new technologies [2]. Although the use of Information Technologies in the healthcare sector in Türkiye is widespread, the use of advanced systems is still limited. There are a total of 33 hospitals using HIMSS in 19 provinces in Türkiye. However, only two public hospitals have adopted HIMSS-7. One of these hospitals is in Istanbul, while the other is in Izmir, a different province [6].

The use of HIMS allows nurses to manage service inputs, working hours, and shift schedules; facilitates documentation processes; ensures that the nursing process is maintained in a computerized environment; enables nurses to spend more time with pediatric patients; and contributes to the improvement and development of child health by supporting the provision of holistic professional care services [7].

Studies have indicated that the adoption of HIMS reduces medication errors by 55%, shortens procedural and patient admission times by 36% [8], increases physician satisfaction by 13% [9], boosts the use of clinical decision support by 69%, improves patient safety by 65%, and enhances staff satisfaction by 60% [10]. The use of HIMS in intensive care units in Türkiye has been shown to save an average of 95 min per 12-hour shift for nurses [11], reduce patient admission times by 20%, and improve inventory management by 15% thanks to HIMS integration [12]. The integration of HIMS into consultation processes has resulted in faster and more effective execution of these processes [13].

The use of hospital information systems may cause a type of stress known as techno-stress among healthcare professionals. “Techno-stress refers to the negative emotions, such as anxiety, feelings of inadequacy, and increased workload experienced by individuals during the process of adapting to technology” [14]. In recent years, while the widespread use of hospital information systems (HIS) has facilitated the work processes of healthcare professionals, it has also introduced new stressors. A study conducted in Iran by Keshavarz et al. reported that 41% of healthcare professionals experienced moderate levels of techno-stress, 36% experienced high levels, and 23% experienced low levels. In this context, factors such as technological uncertainty, overload, complexity, insecurity, and digital invasion have been identified as the main triggers of techno-stress [15]. Hospitals are organizations within the service sector, and nurses working in hospital settings often face challenges such as irregular working hours and days, low wages, and hospital policies, all of which may affect job satisfaction. However, the integration of technological developments into nursing services appears to have a significant positive impact on job satisfaction. “Job satisfaction is defined as the sense of fulfillment a person derives from their work when the characteristics of the job align with their professional expectations.” In the study by Al Maqbali et al., techno-stress was found to negatively affect nurses’ job satisfaction, while the adoption of HIMS had a positive effect on job satisfaction [16]. Wirkkala et al. found a significant correlation between technology-related stress and emotional exhaustion [17]. A study carried out by Mohamed et al. in Somalia found that the use of appropriate digital systems contributed positively to work productivity and increased employee satisfaction [18]. Keshavarz et al. investigated the techno-stressors experienced by healthcare professionals in a hospital setting and found that those who had worked with the HIMS for a shorter period experienced higher levels of techno-stress compared to long-term users [15]. Stadin et al. examined the handling strategies of healthcare professionals with techno-stress and found that those who perceived HIMS as a convenience had lower levels of techno-stress [19].

There are studies in the literature on HIMSS [20–22], HIMS 7 [23–26], techno stress [27–32], and job satisfaction [28, 30, 32]. However, the number of these studies is limited. Therefore, this study aims to contribute to the literature and evidence-based nursing practices.

Some serious medication errors can be significantly reduced by 55% through.

the use of computerized physician entry system (Bates et al. 1998, pp. 1311–1316).

## Methods

### Objective of the study

This study aimed to find the effects of HIS adoption on techno-stress and job satisfaction of nurses working in pediatric clinics. Study searched for answers to the following questions: “Does HIS adoption act on techno-stress?” and “Does HIS adoption act on job satisfaction?”

### Type of study

The study is a descriptive, correlational study. The study was conducted in a public hospital in Istanbul between January-December 2022.

## Study population and sample

The study population consisted of 280 nurses; who were currently working in the pediatric clinics of a public hospital in Istanbul, and who were currently using the HIMSS-7 system. The study sample consisted of 190 nurses, who volunteered to participate in the study. The sample size was calculated using the equation published by Salant and Dillman [33]. Using this equation, the required sample size for this non-homogeneous population was found to consist of 162 nurses with a 95% confidence interval and ± 5% sampling error as shown below: n = *280 (1.96)*^*2*^ *(0.5) (0.5) / (0.5)*^*2*^ *(280-1) + (1.96)*^*2*^ *(0.5) (0.5) =* 162.

The study included 190 nurses.

## Data collection method

The study data were collected by questionnaire method. The questionnaires were collected by face-to-face interviews since the sample group was accessible. The data were obtained by interviewing nurses working in the neonatal intensive care unit, pediatric service 1, pediatric service 2, pediatric emergency units of the hospital where the study was conducted. The nurses who volunteered to participate in the study were collected after the necessary preliminary information was given and written informed consent was obtained. Data were collected by two researchers during working hours. It took approximately 30 min to collect data from a volunteer.

### Inclusion criteria

Working in pediatric clinics.

Volunteering to participate in the research.

Using HIMS-7.

### Exclusion criteria

Not having received HIMS 7 orientation training.

## Data collection tools

The Demographic Information Form”, “Hospital Information Systems Adoption Scale”, “Techno-stress Scale”, and “Minnesota Job Satisfaction Questionnaire” were used for data collection.

### Demographic information form

This questionnaire consisted of 8 questions about the demographic characteristics of nurses, who were currently using HIMSS, and who agreed to participate in the study.

### Hospital information systems adoption scale (HISAS)

This tool was developed by Venkatesh et al. [34] and the validity and reliability study of the scale in Turkish was conducted by Engin and Gürses [35]. The scale consists of six sub-dimensions and a total of 21 items. Sub-dimensions; Performance expectancy, Effort/effort expectancy, Social influence, Facilitating conditions, Behavioral intention, Utilization. The scale is a 7-point Likert-type scale. There are 7 answer options from 1 - strongly disagree to 7 - strongly agree. A high score indicates that the scale is reliable and HIS has been adopted. In that study, Cronbach’s Alpha value for the HISAS was high and reported as 0.937. In our study, Cronbach’s Alpha value for the scale was high, too, and it was 0.851.

### Techno-stress scale

This 25-question scale was developed by Tarafdar, Tu, Ragu-Nathan, and Ragu-Nathan in 2007 to examine the effects of technological changes on employees. The scale consisted of the following five sub-dimensions: “techno-overload”, “techno-invasion”, “techno-complexity”, “techno-insecurity”, and “techno-uncertainty”. Cronbach’s alpha values of these sub-dimensions were reported as 0.89, 0.81, 0.84, 0.84, and 0.82, respectively. In the study on aviation sector employees in 2015, Alam simplified the scale as a short form, consisting of 14 questions and three following sub-dimensions: “techno-overload”, “techno-complexity”, and “techno-uncertainty”. The scale is a 5-point Likert-type scale. There are 5 answer options ranging from 1-strongly disagree to 5-strongly agree.

High scores indicated an increase in the techno-stress level of the individual. Alpha values reported by Alam [36] were 0.83, 0.71, and 0.78, respectively. The validity and reliability study of this 14-question scale in Turkish was conducted by Türen, Erdem, and Kalkın [37]. In this study, the Cronbach’s Alpha value of the Techno Stress Scale was high and it was 0.90.

### Minnesota job satisfaction questionnairee

Questionnaire was developed by Weiss, Dawis, England, and Lofquist [38] to evaluate employees’ job satisfaction. The questionnaire has a two-factor structure: intrinsic satisfaction and extrinsic satisfaction. The score obtained from the entire questionnaire determines the general satisfaction level. ***Intrinsic satisfaction*** is a measure of qualities such as independence, social status, moral values, authority, and ability utilization. ***Extrinsic satisfaction*** is a measure of qualities such as supervision, company policies, recognition, and working conditions. The original study reported the Cronbach’s alpha value of the questionnaire as 0.94. The validity and reliability study of the Short-Form Minnesota Satisfaction Questionnaire (SF-MSQ) in Turkish was conducted by Baycan [39]. This questionnaire consists of 20 items to be scored on a 5-point Likert-type scale. The intrinsic and extrinsic satisfaction subscales consist of 12 and 8 items, respectively. Job satisfaction is considered to increase as it approaches the maximum score, which can be calculated as the average or the total score of the questionnaire. Cronbach’s alpha value was reported as 0.88. In this study, the reliability of the Job Satisfaction scale was found to be high with a Cronbach’s Alpha value of 0.81.

## Statistical analysis of data

Study data were analyzed in a computer environment by using the SPSS 22.0 statistical software. Frequency and percentage were used to examine the demographic characteristics. Mean and standard deviation were used to examine the scale or questionnaire scores. Correlations across the dimensions of the measurement tool scores of nurses were examined by Pearson’s correlation analysis and linear regression analyses. Correlations were considered to be of the very weak, weak, moderate, high, and very high qualities when correlation coefficients (r) were in the ranges of 0.00-0.25, 0.26–0.49, 0.50–0.69, 0.70–0.89, and 0.90-1.00, respectively [40]. The t-test, one-way analysis of variance (ANOVA), and post hoc analyses (Tukey-LSD) were used to examine the differences in measurement tool scores by the demographic characteristics of nurses. Cohen’s d and Eta-squared (η^2^) coefficients were used to calculate the effect size, which would indicate whether the difference between groups was large enough to be considered significant. In the literature, for Cohen’s d values, a value of 0.2 is considered small, 0.5 is considered moderate, and 0.8 is considered large, and for eta-squared values, a value of 0.01 is considered small, 0.06 is considered moderate, and 0.14 is considered large [41].

### Ethics approval and consent to participate

In order to carry out the study, the ethics committee approval was obtained from Istanbul Medipol University’s Non-Interventional Clinical Research Ethics Committee (Number: E-10840098-772.02-2164, Date: 04.04.2022, Decision Number:300) and the permission for the study conduct was obtained from the Istanbul Provincial Health Directorate. Participants were informed of the aims and procedures of the study. Written consent was obtained from the participants stating that they agreed to participate in the research. Measures were taken to protect the confidentiality and anonymity of nurses.Data confidentiality was maintained throughout the study. Participants were informed that they had the right to refuse participation or withdraw from the study at any point. The Declaration of Helsinki was adhered to throughout the study.

## Results

Of the nurses participating in the study, 68.9% were women, 46.8% were in the age range of 18–24 years, 62.1% were single, and 66.3% had a bachelor’s degree or higher. Table 1 shows the demographic characteristics of the participants.

**Table 1:** Demographic characteristics (*N* = 190)

Groups		Frequency (*n*)	Percentage (%)
Gender	Women	**131**	**68.9**
	Men	59	31.1
Age (years)	18–24	**89**	**46.8**
	25–34	83	43.7
	≥ 35	18	9.5
Marital status	Married	72	37.9
	Single	**118**	**62.1**
Educational status	High school	43	22.6
	Associate degree	21	11.1
	Bachelor’s degree and higher	**126**	**66.3**
Income	Income less than expenses	23	12.1
	Income equal to expenses	46	24.2
	Income higher than expenses	**121**	**63.7**
Working experience	0–3 years	**83**	**43.7**
	4–10 years	67	35.3
	> 11 years	40	21.1
Professional experience	0–3 years	**91**	**47.9**
	3–10 years	78	41.1
	> 10 years	21	11.1
Experience with computer usage	0–3 years	32	16.8
	3–10 years	**117**	**61.6**
	> 10 years	41	21.6
HIS usage experience	0–3 years	**104**	**54.7**
	3–10 years	39	20.5
	> 11 years	47	24.7

Nurses’ mean score from the HISAS was high (5.78 ± 0.68), mean overall score from the Techno-Stress Scale was moderate (3.29 ± 0.80), and mean overall score from the job satisfaction questionnaire was high (3.22 ± 0.40) (Table 2).

**Table 2:** Mean scores for hospital information systems adoption, techno-stress, and job satisfaction (*N* = 190)

Measurement Tool	Sub-dimension	Mean ± SD	Min.	Max.
HISAS	Performance expectancy	5.71 **±** 1.01	2.75	7.00
	Effort expectancy	5.75 **±** 1.04	3.25	7.00
	Social influence	5.98 **±** 0.50	4.33	7.00
	Facilitating conditions	5.97 **±** 0.59	4.40	7.00
	Behavioral intention	5.37 **±** 1.61	1.67	7.00
	Use	5.79 **±** 0.75	3.00	7.00
	**Overall**	**5.78 ± 0.68**	**4.05**	**6.76**
Techno-stress	Techno-overload	3.38 **±** 1.13	1.20	5.00
	Techno-complexity	3.49 **±** 0.72	1.60	4.60
	Techno-uncertainty	2.93 **±** 0.86	1.25	4.50
	**Overall**	**3.29 ± 0.80**	**1.64**	**4.64**
Job satisfaction	Intrinsic satisfaction	3.67 **±** 0.36	2.75	4.33
	Extrinsic satisfaction	2.54 **±** 0.62	1.25	3.38
	**Overall**	**3.22 ± 0.40**	**2.40**	**3.85**

The regression analysis for the examination of the relationship between the overall scores from the Techno-Stress Scale and the overall and sub-dimension scores from the HISAS revealed statistical significance (*p* = 0.00). We found that adoption of HIS reduced the level of techno-stress (Table 3).

**Table 3:** The effect of hospital information system adoption on techno-stress (*N* = 190)

Dependent variable	Independent variable	ß	t	*p*	F	Model (*p*)	*R* ^2^
Techno-stress overall	Fixed	8.874	31.350	**0.000**	393.548	**0.000**	0.675
	Overall adoption of hospital information systems	-0.964	-19.838	**0.000**			
Techno-stress overall	Fixed	7.868	12.970	**0.000**	70.410	**0.000**	0.688
	Performance expectancy	-0.177	-3.016	**0.003**			
	Effort expectancy	-0.130	-2.398	**0.017**			
	Social influence	-0.069	-0.996	0.321			
	Facilitating conditions	-0.048	-0.682	0.496			
	Behavioral intention	-0.240	-6.191	**0.000**			
	Use	-0.141	-3.083	**0.002**			

Linear regression analysis

The correlation analysis revealed negative and high correlations between the overall score of the Techno-Stress Scale and the overall scores (*r* = -0.823, *p* = 0.000), performance expectancy scores (*r* = -0.738, *p* = 0.000), effort expectancy scores (*r* = -0.73, *p* = 0.000), and behavioral intention scores (*r* = -0.798, *p* = 0.000) of the HISAS. There was a negative and weak correlation between the overall score of the Techno-Stress Scale and the social influence score (*r* = -0.301, *p* = 0.000) and there was a negative and moderate correlation between the overall score of the Techno-Stress Scale and the facilitating conditions score (*r* = -0.525, *p* = 0.000).

The overall scores of job satisfaction were positively and highly correlated with the overall scores (*r* = 0.838, *p* = 0.000), performance expectancy scores (*r* = 0.753, *p* = 0.000), effort expectancy scores (*r* = 0,772, *p* = 0.000), and behavioral intention scores (*r* = 0.797, *p* = 0.000) of the HISAS. The correlation between the overall score of job satisfaction and the social influence score was positive and very weak (*r* = 0.218, *p* = 0.003) and the correlation between the overall score of job satisfaction and the facilitating conditions score was positive and moderate (*r* = 0.578, *p* = 0.000).

The regression analysis for the examination of the relationship between the overall scores from the Job Satisfaction Questionnaire and the overall and sub-dimension scores from the HISAS revealed statistical significance (*p* = 0.000). We found that adoption of HIS increased the level of job satisfaction (Table 4).

**Table 4:** The effect of hospital information system adoption on job satisfaction (*N* = 190)

Dependent variable	Independent variable	ß	t	*p*	F	Model (*p*)	*R* ^2^
Job satisfaction overall	Fixed	0.347	2.519	**0.013**	441.641	**0.000**	0.700
	Overall adoption of hospital information systems	0.497	21.015	**0.000**			
Job satisfaction overall	Fixed	1.197	4.064	**0.000**	79.224	**0.000**	0.713
	Performance expectancy	0.089	3.124	**0.002**			
	Effort expectancy	0.107	4.068	**0.000**			
	Social influence	-0.039	-1.172	0.243			
	Facilitating conditions	0.059	1.721	0.087			
	Behavioral intention	0.098	5.197	**0.000**			
	Use	0.044	1.979	**0.049**			

Linear Regression Analysis

There were significant correlations between the HIS adoption scores of the nurses participating in the study and the nurses’ age, marital status, educational status, income levels, working experience, professional experience, and HIS usage experience (*p* = 0.000). The scores of adoption of HIS were high among nurses, who were in the age range of 18–24 years, who were single, who had a bachelor’s degree or above, and whose income was equal to their expenses. Furthermore, compared to other groups, HIS adoption scores were higher among nurses, who had 0–3 years of working experience and more than 10 years of professional experience and HIS usage experience.

There were significant correlations between the techno-stress scores of the participating nurses and the nurses’ age, marital status, educational status, income levels, working experience, professional experience, computer use experience, and HIS usage experience (*p* < 0.05). Techno-stress levels were high among nurses, who were 35 years of age and over, who were married, and who were high school graduates, and those whose income was higher than their expenses. When compared by experience levels, we found increased techno-stress levels among nurses with 3–10 years of working experience, professional experience, and HIS usage experience, and among nurses with 0–3 years of computer usage experience.

We found a significant difference between nurses’ job satisfaction scores and their age, marital status, educational status, income levels, working experience, professional experience, and HIS usage experience (*p* < 0.05). Job satisfaction scores were high among nurses, who were in the age range of 18–24 years, who were single, who had a bachelor’s degree or above, and whose income was equal to their expenses. Job satisfaction scores were also high among nurses, who had been working for 0–3 years and who had more than 10 years of professional and HIS usage experience (Table 5).

**Table 5:** Comparison of mean scores from the HISAS, Techno-Stress scale, and job satisfaction questionnaire by demographic characteristics (*N* = 190)

Characteristics		HIS adoption		Techno-stress	Job satisfaction
		n	Mean ± SD	Mean ± SD	Mean ± SD
Gender	Women	131	5.77 ± 0.69	3.29 ± 0.82	3.22 ± 0.41
	Men	59	5.79 ± 0.67	3.30 ± 0.74	3.21 ± 0.39
	t=		-0.151	-0.117	0.264
	p=		0.880	0.907	0.792
Age (years)	18–24	89	6.12 ± 0.48	3.11 ± 0.46	3.36 ± 0.28
	25–34	83	5.43 ± 0.71	3.45 ± 1.01	3.10 ± 0.46
	≥ 35	18	5.68 ± 0.60	3.47 ± 0.84	3.05 ± 0.41
	F=		28.269	4.459	10.978
	p=		**0.000**	**0.013**	**0.000**
	Post Hoc=		1 > 2, 1 > 3 (*p* < 0.05)	2 > 1 (*p* < 0.05)	1 > 2, 1 > 3 (*p* < 0.05)
Marital status	Married	72	5.54 ± 0.74	3.51 ± 0.85	3.11 ± 0.42
	Single	118	5.92 ± 0.60	3.16 ± 0.74	3.29 ± 0.38
	t=		-3.822	2.907	-2.997
	p=		**0.000**	**0.006**	**0.004**
Educational Status	High school	43	4.79 ± 0.22	4.36 ± 0.18	2.67 ± 0.13
	Associate degree	21	5.93 ± 0.52	2.92 ± 0.84	3.35 ± 0.34
	Bachelor’s degree and higher	126	6.09 ± 0.46	2.99 ± 0.58	3.38 ± 0.30
	F=		148.545	101.322	105.973
	p=		**0.000**	**0.000**	**0.000**
	Post Hoc=		2 > 1, 3 > 1 (*p* < 0.05)	1 > 2, 1 > 3 (*p* < 0.05)	2 > 1, 3 > 1 (*p* < 0.05)
Income	Income less than expenses	23	5.65 ± 0.74	3.48 ± 0.71	3.13 ± 0.38
	Income equal to expenses	46	6.13 ± 0.28	2.59 ± 0.67	3.46 ± 0.29
	Income higher than expenses	121	5.67 ± 0.73	3.52 ± 0.70	3.15 ± 0.41
	F=		8.559	30.415	11.498
	p=		**0.000**	**0.000**	**0.000**
	Post Hoc=		2 > 1, 2 > 3 (*p* < 0.05)	1 > 2, 3 > 2 (*p* < 0.05)	2 > 1, 2 > 3 (*p* < 0.05)
Working experience	0–3 years	83	6.10 ± 0.47	3.10 ± 0.47	3.39 ± 0.26
	3–10 years	67	5.19 ± 0.68	3.92 ± 0.76	2.91 ± 0.40
	> 10 years	40	6.08 ± 0.30	2.63 ± 0.67	3.38 ± 0.35
	F=		62.468	58.648	44.020
	p=		**0.000**	**0.000**	**0.000**
	Post Hoc=		1 > 2, 3 > 2 (*p* < 0.05)	2 > 1, 1 > 3, 2 > 3 (*p* < 0.05)	1 > 2, 3 > 2 (*p* < 0.05)
Professional experience	0–3 years	91	6.07 ± 0.49	3.09 ± 0.50	3.38 ± 0.28
	3–10 years	78	5.35 ± 0.68	3.75 ± 0.79	2.95 ± 0.38
	> 10 years	21	6.10 ± 0.58	2.49 ± 0.90	3.50 ± 0.36
	F=		35.590	35.744	41.563
	p=		**0.000**	**0.000**	**0.000**
	Post Hoc=		1 > 2, 3 > 2 (*p* < 0.05)	2 > 1, 1 > 3, 2 > 3 (*p* < 0.05)	1 > 2, 3 > 2 (*p* < 0.05)
Experience with computer usage	0–3 years	32	5.70 ± 0.77	3.54 ± 0.74	3.12 ± 0.38
	3–10 years	117	5.75 ± 0.70	3.37 ± 0.76	3.21 ± 0.41
	> 10 years	41	5.93 ± 0.53	2.87 ± 0.81	3.33 ± 0.36
	F=		1.322	8.410	2.725
	p=		0.269	**0.000**	0.068
	Post Hoc=			1 > 3, 2 > 3 (*p* < 0.05)	
HIS usage experience	0–3 years	104	5.86 ± 0.65	3.33 ± 0.65	3.25 ± 0.37
	3–10 years	39	5.29 ± 0.68	3.82 ± 0.78	2.95 ± 0.39
	> 10 years	47	6.00 ± 0.54	2.77 ± 0.81	3.36 ± 0.38
	F=		15.359	22.997	13.459
	p=		**0.000**	**0.000**	**0.000**
	Post Hoc=		1 > 2, 3 > 2 (*p* < 0.05)	2 > 1, 1 > 3, 2 > 3 (*p* < 0.05)	1 > 2, 3 > 2 (*p* < 0.05)

F: ANOVA test; t: Independent Groups t-Test; Post Hoc: Tukey-LSD

It was determined that there was a significant difference between the technological burden sub-dimension scores of the nurses participating in the study and marital status, educational status, income level, working experience, professional experience, and experience of using HBS (*p* < 0.05). It was determined that there was a significant difference between the technological complexity sub-dimension scores of the nurses and educational status, income level, working experience, professional experience, computer experience, experience of using HBS (*p* < 0.05). It was found that there was a significant difference between technological uncertainty subscale scores and age, marital status, educational status, income level, working experience, professional experience, computer experience and experience of using HBS (*p* < 0.05), (Table 6).

The technological uncertainty scores of nurses in the 25–34 age range were found to be high. Married nurses had high technological burden and technological uncertainty scores. High school graduates had high scores in all sub-dimensions. Those with low income level had high technological burden scores, while those with high income level had high technological complexity and technological uncertainty scores. Those with 3–10 years of work experience, professional experience and experience in the use of HBS had higher sub-dimension scores than the other groups. In addition, those with 0–3 years of computer experience had higher technological complexity scores, while those with 3–10 years of computer experience had higher technological uncertainty scores (Table 6).

**Table 6:** Differentiation of techno stress scores according to descriptive characteristics

Characteristics		*n*	Techno-overload	Techno-complexity	Techno-uncertainty
			Ort ± SS	Ort ± SS	Ort ± SS
Gender	Women	131	3,350 ± 1,165	3,495 ± 0,760	2,970 ± 0,873
	Men	59	3,468 ± 1,075	3,498 ± 0,661	2,869 ± 0,844
	t=		-0,662	-0,032	0,744
	p=		0,508	0,975	0,458
Age (years)	18–24	89	3,207 ± 0,885	3,429 ± 0,506	2,610 ± 0,542
	25–34	83	3,513 ± 1,318	3,559 ± 0,906	3,247 ± 0,990
	≥ 35	18	3,689 ± 1,254	3,533 ± 0,761	3,139 ± 0,979
	F=		2,300	0,705	13,936
	p=		0,103	0,495	**0**,**000**
	Post Hoc=				2 > 1, 3 > 1 (*p* < 0.05)
Marital status	Married	72	3,633 ± 1,142	3,628 ± 0,765	3,208 ± 0,922
	Single	118	3,236 ± 1,111	3,415 ± 0,697	2,773 ± 0,784
	t=		2,370	1,964	3,468
	p=		**0**,**019**	0,051	**0**,**001**
Educational Status	High school	43	4,554 ± 0,447	4,307 ± 0,211	4,198 ± 0,270
	Associate degree	21	3,095 ± 1,356	2,991 ± 0,887	2,619 ± 0,714
	Bachelor’s degree and higher	126	3,037 ± 0,992	3,303 ± 0,597	2,562 ± 0,560
	F=		42,136	57,691	157,181
	p=		**0**,**000**	**0**,**000**	**0**,**000**
	Post Hoc=		1 > 2, 1 > 3 (*p* < 0.05)	1 > 2, 3 > 2, 1 > 3 (*p* < 0.05)	1 > 2, 1 > 3 (*p* < 0.05)
Income	Income less than expenses	23	3,713 ± 1,078	3,565 ± 0,626	3,087 ± 0,906
	Income equal to expenses	46	2,804 ± 1,365	2,670 ± 0,578	2,245 ± 0,314
	Income higher than expenses	121	3,546 ± 0,972	3,797 ± 0,535	3,174 ± 0,861
	F=		8,853	68,476	24,635
	p=		**0**,**000**	**0**,**000**	**0**,**000**
	Post Hoc=		1 > 2, 3 > 2 (*p* < 0.05)	1 > 2, 3 > 2 (*p* < 0.05)	1 > 2, 3 > 2 (*p* < 0.05)
Working experience	0–3 years	83	3,029 ± 0,747	3,516 ± 0,417	2,696 ± 0,544
	3–10 years	67	4,027 ± 1,008	4,018 ± 0,641	3,687 ± 0,860
	> 10 years	40	3,055 ± 1,508	2,580 ± 0,432	2,188 ± 0,264
	F=		19,719	99,717	80,142
	p=		**0**,**000**	**0**,**000**	**0**,**000**
	Post Hoc=		2 > 1, 2 > 3 (*p* < 0.05)	2 > 1, 1 > 3, 2 > 3 (*p* < 0.05)	2 > 1, 1 > 3, 2 > 3 (*p* < 0.05)
Professional experience	0–3 years	91	3,075 ± 0,834	3,470 ± 0,505	2,640 ± 0,564
	3–10 years	78	4,056 ± 1,046	3,741 ± 0,763	3,391 ± 0,972
	> 10 years	21	2,248 ± 1,140	2,695 ± 0,850	2,548 ± 0,777
	F=		38,457	20,699	22,490
	p=		**0**,**000**	**0**,**000**	**0**,**000**
	Post Hoc=		2 > 1, 1 > 3, 2 > 3 (*p* < 0.05)	2 > 1, 1 > 3, 2 > 3 (*p* < 0.05)	2 > 1, 2 > 3 (*p* < 0.05)
Experience with computer usage	0–3 years	91	3,075 ± 0,834	3,470 ± 0,505	2,640 ± 0,564
	3–10 years	78	4,056 ± 1,046	3,741 ± 0,763	3,391 ± 0,972
	> 10 years	21	2,248 ± 1,140	2,695 ± 0,850	2,548 ± 0,777
	F=		38,457	20,699	22,490
	p=		**0**,**000**	**0**,**000**	**0**,**000**
	Post Hoc=		2 > 1, 1 > 3, 2 > 3 (*p* < 0.05)	2 > 1, 1 > 3, 2 > 3 (*p* < 0.05)	2 > 1, 2 > 3 (*p* < 0.05)
HIS usage experience	0–3 years	32	3,781 ± 1,061	3,706 ± 0,616	3,047 ± 0,874
	3–10 years	117	3,357 ± 1,015	3,634 ± 0,679	3,083 ± 0,865
	> 10 years	41	3,161 ± 1,435	2,937 ± 0,683	2,439 ± 0,654
	F=		2,832	18,348	9,565
	p=		0,061	**0**,**000**	**0**,**000**
	Post Hoc=			1 > 3, 2 > 3 (*p* < 0.05)	1 > 3, 2 > 3 (*p* < 0.05)
HIS usage experience	0–3 years	104	3,350 ± 0,939	3,650 ± 0,511	2,928 ± 0,780
	3–10 years	39	3,851 ± 1,043	3,954 ± 0,645	3,635 ± 0,819
	> 10 years	47	3,081 ± 1,465	2,775 ± 0,701	2,383 ± 0,644
	F=		5,244	50,237	29,118
	p=		**0**,**006**	**0**,**000**	**0**,**000**
	Post Hoc=		2 > 1, 2 > 3 (*p* < 0.05)	2 > 1, 1 > 3, 2 > 3 (*p* < 0.05)	2 > 1, 1 > 3, 2 > 3 (*p* < 0.05)

F: Anova Testi; t: Bağımsız Gruplar T-Testi; PostHoc: Tukey, LSD

## Discussion

This study shows that HIS adoption is negatively related to techno-stress and positively related to job satisfaction among nurses. As nurses’ level of HIS adoption increases, the level of techno-stress decreases and the level of job satisfaction increases.

The mean HIS adoption score of the participants is above average. Along with rapidly ongoing advances in technology so far, studies from our country show that the opinions of healthcare professionals on HIS usability and adoption have become more positive over the years compared to the past [42]. Furthermore, the use of HIMMS Level 7 in the hospital, where this study was conducted, appears to have favorably affected the adoption of HIS.

In hospitals that use the HIMS 7 system, all data, documents, and medical images are processed together within the Electronic Medical Record (EMR) environment. Digitally stored data is actively used to analyze clinical data models/templates to improve the quality of healthcare services, ensure patient safety, and deliver effective care. Clinical information may be easily shared, when necessary, with all organizations and individuals authorized to treat the patient via standardized electronic processes (CCD). These conveniences provided by the system to the employees are believed to positively affect the adoption of HIMS by nurses [43].

In this study, the mean scores were higher than the moderate level in the following sub-dimensions of the HISAS, including Performance Expectancy, Effort Expectancy, Social Influence, Facilitating Conditions, Behavioral Intention, and Use Behavior, with the Social Influence and Facilitating Conditions sub-dimensions having the highest mean scores. A study investigating the factors acting on the HIS adoption status of 392 healthcare professionals in Turkey Bursa and Balıkesir reported that the variables of Performance Expectancy, Effort Expectancy, and Social Influence positively affected Behavioral Intention for HIS usage, and the variables Facilitating Conditions and Behavioral Intention had a positive effect on Use Behavior [35]. A study, which included 400 healthcare professionals in Iran, reported that the variables, Performance Expectancy, Effort Expectancy, and Social Influence had a positive effect on Behavioral Intention for HIS usage, and Facilitating Conditions and Behavioral Intention factors had a positive effect on Use Behavior [44]. Another study examining the HIS adoption status of 660 nurses in Ghana reported that Social Influence and Facilitating Conditions had a significant impact on Behavioral Intention for HIS use [45]. Another study from Taiwan, aiming to evaluate the attitudes of 434 nurses toward mobile learning, reported that Satisfaction, Social Influence, Performance Expectancy, Facilitating Conditions, and Effort Expectancy positively acted on nurses’ behavioral intention to use mobile learning [46]. Studies in the literature show that a variety of variables individually had different impact rates on HIS adoption and usage. This may suggest the role of differences in institutional culture across individual hospitals.

Techno-stress is described as an individual’s response to advances and changes in technology. Today, technology is actively used in the healthcare sector, as in many other sectors [31]. In this study, we have found that the participants’ overall mean techno-stress scores are moderate, with the highest mean score obtained from the Techno-Complexity sub-dimension. Studies are available in the literature, showing that nurses’ techno-stress levels were observed at moderate levels [31, 47].

In general, consistent with our results, studies in the literature report mean techno-stress scores at moderate or close to moderate levels. We believe that techno-stress levels of personnel may increase in parallel with the increase in the digitalization level of hospitals.

The healthcare sector is distinguished by the intense professional efforts of personnel. This suggests that effective healthcare services mostly depend on the performance of healthcare professionals and, thus, their levels of job satisfaction [48]. In this study, we found that the mean total scores of job satisfaction among nurses were at a moderate level. We think that employees’ job satisfaction may vary depending on individual and institutional factors.

In this study, we have found a significantly high and negative correlation between HIS Adoption and techno-stress, and a significantly high and positive correlation between HIS Adoption and job satisfaction. As the level of HIS adoption decreases, the level of techno-stress increases, and the level of job satisfaction decreases. The regression analysis on the impact of HIS adoption on techno-stress and job satisfaction revealed that 67.5% of the change in the general level of techno-stress and 70% of the change in the general level of job satisfaction could be explained by HIS adoption. Our study results show that HIS adoption and acceptance are highly effective in the alleviation of techno-stress and enhancement of job satisfaction.

Gaube et al., in their study on 445 healthcare professionals in Germany, examined the relationship between the perception of health information technology features and psychological and organizational variables. They found that better health information technology use was associated with lower techno-stress levels, and the use of information technologies was associated with less strain. Moreover, the study reported that techno-stress and information technology-related strain were associated with low levels of job satisfaction [49]. The results of a study involving a total of 616 nurses from four different hospitals in Canada showed that Performance Expectancy was the strongest driver in the use of electronic health records, while Social Influence had the most significant overall effect on actual use of the electronic health record [50].

Our study findings support the results reported by studies from Turkey and other countries. However, a literature review reveals that the number of studies holistically examining the interaction of effects of HIS adoption, techno-stress, and job satisfaction among nurses is only a few. This confirms the originality of our study, suggesting that it will contribute to the literature on this subject matter.

In recent years, while the widespread use of hospital information systems (HIS) has facilitated the work processes of healthcare professionals, it has also introduced new stressors. It is also emphasized that effective integration of digital health technologies may improve the performance of healthcare professionals and reduce their workload. In particular, a study conducted in Somalia found that the use of appropriate digital systems contributed positively to work productivity and improved employee satisfaction [18].

The level of HIS adoption and user experience is shaped not only by technological factors but also by cultural and regional contexts. Recent studies have shown that, in particular, power distance, uncertainty avoidance, and cultural structure have a direct impact on the acceptance and use of HIS technologies [51, 52]. However, environmental factors such as infrastructure availability, local health policies, regional economic conditions, and the level of digital literacy may either facilitate or hinder the use of HIS [53].

These findings reveal that HIS adoption presents both opportunities and risks; thus considering the cultural and organizational contexts during system integration processes is critical. Conducting this study in Türkiye ensures that the findings offer meaningful insights within the local context. However, it should be noted that the adoption of HIS—as well as phenomena such as techno-stress and job satisfaction—may vary across different cultural and regional contexts. Our study findings are consistent with the results of previous studies conducted both in Türkiye and other countries.

We have found that, compared to other groups of participants in our study, HIS adoption levels were higher among nurses, who were in the age range of 18–24, who were single, who had a bachelor’s degree or above, whose income was equal to their expenses, who had 0–3 years of working experience, and who had more than 10 years of professional and HIS usage experience.

Research shows that nurses’ educational background affects their attitudes toward information technologies. Strudwick reported that nurses with postgraduate education or higher used electronic health record systems more effectively and expressed more positive opinions regarding their contribution to patient care [54]. Similarly, Eriş reported that as the education level of nurses increased, both the frequency of HIMS use and satisfaction with these systems also significantly increased [55].

The professional experience level of nurses may directly affect their behavior in adopting and using health information systems. A study by Alrasheeday et al. in Saudi Arabia found that nurses with greater professional experience were more cautious and less motivated to use computers [56].

Age emerges as a significant variable influencing attitudes toward technology. A systematic review by Kuek and Hakkennes revealed that younger nurses (aged 20–30) tended to have more positive attitudes toward health information technologies and adopted these systems more readily [57]. Although some studies suggest that gender may influence the use of health information systems, the results are inconsistent. Ismail et al. found that female nurses experienced higher satisfaction and less technological uncertainty in using HIMS than their male colleagues [58].

It has been reported that individuals with a bachelor’s degree obtain lower HIS adoption scores compared to other educational degrees, and those with a working experience of 10 years or longer obtain higher HIS adoption scores compared to their counterparts. De Veer et- al. Netherlands evaluated nurses’ attitudes toward EHCR use. That study reported no differences in attitude scores toward EHCR use by the variables of age, gender, or marital status; however, nurses with a bachelor’s degree or above and nurses with 6–10 years of professional experience had more positive attitudes toward EHCR use [59].

In our study, we found increased techno-stress levels among nurses, who were 35 years of age and over, married, and high school graduates, and whose income was higher than their expenses. Our comparison by experience levels showed increased techno-stress levels among nurses with 3–10 years of working experience, professional experience, and HIS usage experience, and among nurses with 0–3 years of computer usage experience. In their study conducted in Türkiye, Gürdaş Topkaya & Kaya found significant differences between nurses’ ages and their attitudes toward computer use. Nurses, especially those between the ages of 25–34, demonstrated more positive attitudes toward technology [60]. Similarly, a study carried out by Saray and Ünsal in Türkiye found that nurses with 3–10 years of professional experience exhibited more favorable attitudes toward technology than other groups [61].

Regarding technology subgroups, our study found that technological uncertainty scores in nurses aged 25–34, technological overload and uncertainty scores in married nurses, all subdimension scores in high school graduates, technological overload scores in nurses with low incomes, and technological overload and uncertainty scores in nurses with high incomes were higher. The subdimension scores of those with 3–10 years of work, professional, and HIS experience were higher compared to the other groups. Furthermore, nurses with 0–3 years of computer experience scored higher in technological complexity, while those with 3–10 years of experience scored higher in technological uncertainty.

Kaya et al. found that married nurses perceived higher levels of technological overload and uncertainty, while high school graduate nurses scored higher across all subdimensions of techno-stress [62]. Saray & Ünsal identified a significant correlation between nurses’ income levels and their attitudes toward technology in Türkiye. Low-income nurses demonstrated higher levels of perceived technological overload, whereas high-income nurses reported higher perceptions of technological complexity and uncertainty [61]. Gürdaş Topkaya & Kaya found that nurses with 0–3 years of computer experience had higher perceptions of technological complexity, while those with 3–10 years of experience scored higher on technological uncertainty [60]. Ismail et al. found in their study in Saudi Arabia that more experienced nurses approached technology with greater caution; in other words, as the level of experience increased, attitudes toward computer use decreased [58]. In their study performed in the UK, Brown et al. revealed that nurses’ perceptions of technological uncertainty were associated with demographic factors such as age, gender, and education level, with older and less educated nurses having higher perceptions of uncertainty. They also found that nurses with less experience perceived technology as more complex [63].

Kopuz and Aydın, in their study in Türkiye, reported that healthcare professionals who were female, high school graduates, and declared a high monthly income exhibited higher levels of techno-uncertainty. While the duration of employment in the organization or in the profession did not significantly affect techno-stress levels, it was found that techno-complexity decreased with increasing age [64]. Golz et al., found no difference in techno-stress levels based on gender among healthcare professionals. However, younger individuals experienced lower levels of techno-stress [31].

Golz, et al. reported in their study in Switzerland that mostly physicians, followed by nurses, experienced higher levels of techno-stress than other healthcare professionals, that there was a significant increase in the level of techno-stress with increased levels of education, and that social support played an important role in the alleviation of techno-stress levels [47].

In our study, we found high job satisfaction levels among nurses, who were in the age range of 18–24, who were single, who had a bachelor’s degree or above, whose income was equal to their expenses, who had 0–3 years of working experience, and who had more than 10 years of professional and HIS usage experience.

In a study conducted by Al Maqbadi with nurses, it was reported that gender, marital status, education level, and years of work experience did not significantly affect job satisfaction. However, nurses aged 36–45 and 46–55 had the highest levels of job satisfaction, while those aged 18–25 and 26–35 had the lowest [16].

Al-Dossary et al., in a study with nurses in Saudi Arabia, found that age, gender, and education level did not affect job satisfaction, but there was a significant difference based on nursing experience [65]. Bjørk et al. reported in their study on job satisfaction among nurses in Norway that nurses over the age of 37 were more satisfied than younger nurses, and that those with a master’s degree or who were pursuing further education had higher levels of job satisfaction than those who were not continuing their education [66]. Zurmehly found a strong correlation between education level and job satisfaction, indicating that nurses with a bachelor’s or master’s degree were more satisfied [67].

Curtis investigated the impact of demographic variables on job satisfaction among nurses working in Dublin, Ireland. He found that job satisfaction levels were highest among nurses aged 36–45 and 46–55, and lowest among younger nurses aged 18–25 and 26–35 [68].

Klaus et al. stated that nurses who did not hold a Bachelor of Science in Nursing (BSN) or a higher nursing degree were significantly less satisfied with their jobs [69]. Similarly, Zurmehly found a strong correlation between education level and job satisfaction, reporting that nurses holding a BSN or a master’s degree had higher levels of job satisfaction [67].

Al Maqbali [16] performed a review of 24 studies to investigate the factors affecting nurses’ job satisfaction under two categories as institutional and personal factors. That study reported that job satisfaction was a multi-factorial phenomenon, with the significant impacts of especially cultural and social factors on job satisfaction among nurses from various institutions and countries. Al Maqbali reported that nurses’ job satisfaction or dissatisfaction would vary depending on demographic characteristics such as age, gender, educational status, working experience, and the clinical department as the workplace and added that, despite a vast majority of studies on job satisfaction, no consensus had been reached yet, due to variable working conditions and reasons for job satisfaction or dissatisfaction of nurses across studies. Confirming the results reported by that study, our study results are similar to results reported by some studies, but they are at the same time different from other results reported in the literature.

### Limitations of the study

The study is limited to the only public hospital in Istanbul that uses HIMS 7. Another limitation is that the hospital is a 6-year old institution and the nurse population in the hospital consists of a young population of newly recruited nurses. The study was limited to a total of 190 nurses working in the pediatric clinics of the hospital who volunteered to participate in the study. Organizational, cultural and environmental factors may affect nursing participation and technostress level. The high participation of the female gender in the study constitutes another limitation. The data collected is based solely on the reports received from the participants. An additional limitation is the limited time frame in which this survey was conducted, which may reflect changing emotions. The findings may limit the generalizability of the research. Therefore, repeated and extended studies with a larger sample and with nurses in different hospitals are recommended.

## Conclusion

Among nurses working in pediatric clinics, HIS adoption has been highly and negatively correlated with techno-stress and highly and positively correlated with job satisfaction (*p* < 0.05). As the level of HIS adoption increases, the level of techno-stress decreases and job satisfaction increases. In line with the results obtained from this study; we suggest that regular in-service training about HIS usage needs to be provided for nurses representing the largest professional group among healthcare professionals. Furthermore, HIS usage needs to be widespread for each hospital, along with the implementation of institutional policies developed in accordance with the factors that act favorably on HIS adoption rates among healthcare workers. Finally, there is a need for future large-scale studies on different populations to examine this subject matter further.

## Data Availability

The datasets used and/or analysed during the current study are available from the corresponding author on reasonable request.
